# Understanding Factors Contributing to Vitality in Older Adults: A Qualitative Descriptive Study

**DOI:** 10.3390/geriatrics11040093

**Published:** 2026-07-22

**Authors:** Ilana I. Logvinov, Jennifer Crook, Donna Neff, Cindy Tofthagen, Victoria Loerzel

**Affiliations:** 1 College of Nursing, University of Central Florida, Orlando, FL 32827, USA; 2 Department of Anesthesiology and Perioperative Medicine, Mayo Clinic Florida, Jacksonville, FL 32224, USA; 3 Department of Nursing, Mayo Clinic Florida, Jacksonville, FL 32224, USA

**Keywords:** energy, healthy aging, older adults, vitality, qualitative research

## Abstract

**Background**: By 2030, nearly one-quarter of the U.S. population will be over age 65, emphasizing the need to understand factors that promote healthy aging. Vitality is often reduced to physical indicators in clinical frameworks, overlooking its multidimensional nature. **Methods:** This qualitative descriptive study explored how independently living older adults define and experience vitality, focusing on physical, emotional, cognitive, and social dimensions. Guided by Self-Determination Theory, semi-structured interviews were conducted with 10 participants aged 61–83 years purposively recruited from community and faith-based settings in Northeast Florida. **Results**: Thematic analysis revealed four interconnected themes: (1) *Make Adjustments, Keep Going*; (2) *Being Mentally Jubilant*; (3) *Bonds that Keep Me Going*; and (4) *Thriving Amid Uncertainty*. **Conclusions**: Findings suggest that participants perceived vitality as a dynamic, multidimensional process shaped by adaptation, purpose, and connection rather than physical health alone. These insights underscore the need for holistic approaches to vitality assessment and interventions that support autonomy, competence, and relatedness in later life.

## 1. Introduction

According to the U.S. Census Bureau [1], nearly a quarter of the U.S. population (approximately 73 million people) will be over the age of 65 by 2030, up from 17% in 2020. Although only about 13% of older adults satisfy biomedical definitions of successful aging, nearly half describe themselves as aging successfully when psychosocial factors are included [2]. These differences highlight critiques of traditional successful aging models, which often prioritize biomedical functioning and overlook subjective experiences such as meaning, connection, and psychological vitality. This highlights the need to explore older adults’ perceptions of vitality as a foundation for promoting well-being.

Findings suggest that vitality is a multidimensional state of energy and strength shaped by physical, emotional, cognitive, social, and subjective factors [3,4,5]. These dimensions include physical vitality supporting independence [6,7]; emotional vitality reflecting joy and resilience [8,9]; cognitive vitality involving mental energy maintenance [10]; social vitality involving fulfilling interpersonal relationships [11]; and subjective vitality, an internal sense of aliveness [9,12]. Emotional and social vitality, though experienced internally, are central to overall well-being and connectedness [13].

Current clinical guidelines, however, often emphasize physical health indicators. For example, the Mini Nutritional Assessment (MNA) within the Integrated Care for Older People (ICOPE) vitality domain focuses on nutrition as a proxy for vitality [14], overlooking its broader psychological dimensions. Aging introduces changes in physical health, cognition, relationships, and finances that all shape one’s sense of vitality.

Vitality is inherently subjective, fluctuating with perceived energy [9] and linked to meaning and purpose [12]. Although studies highlight the benefits of physical activity and social engagement [7,15,16], there remains a gap in understanding how older adults themselves define vitality and how their experiences influence it. Older adults’ perspectives offer essential insights into adaptation and well-being. Therefore, the purpose of this study was to explore vitality among independently living older adults through physical, emotional, cognitive, and social lenses and to capture the personal meanings associated with vitality.

To guide the conceptualization of vitality beyond the biomedical indicators, this study draws on Self-Determination Theory (SDT), which emphasizes autonomy, competence, and relatedness as fundamental to human energy and well-being. SDT provides a useful lens for understanding vitality as an internally experienced, psychologically driven construct shaped by individuals’ capacity to adapt, maintain purpose, and remain socially connected in later life. Unlike biomedical or deficit-based models, SDT conceptualizes vitality as a function of psychological need fulfillment, making it particularly suited to understanding how vitality is sustained in later life.

## 2. Methods

### 2.1. Design

A qualitative descriptive study design was selected to explore vitality from participants’ perspective and establish a foundation for future interventions [17,18]. This approach is particularly appropriate for capturing low-inference, practice-relevant insights by staying close to participants’ language and descriptions, rather than imposing higher levels of abstraction. Consistent with principles of naturalistic inquiry, the design allows for the examination of vitality as it is experienced and articulated in real-world contexts. Since ICOPE guidelines are designed to evaluate older adults, a qualitative descriptive methodology was well suited to elicit how older adults define and understand vitality in their own terms.

### 2.2. Theoretical Framework

SDT [19] guided the study’s interview guide and results interpretation, exploring how autonomy [20], competence [20,21], and relatedness [22] influence older adults’ perception of vitality.

### 2.3. Ethical Consideration

The study was conducted in accordance with the Declaration of Helsinki. It was reviewed by the University of Central Florida’s (UCF) Institutional Review Board (IRB) and was determined to qualify for an exemption from IRB review (STUDY00007698) on 26 March 2025. All participants provided verbal consent for participation and audio recording and were informed of measures to ensure confidentiality and secure data storage, as well as their voluntary participation and right to withdraw at any time without penalty.

### 2.4. Researcher Characteristics and Reflexivity

The first author, a novice qualitative researcher, approached the study with an understanding that vitality extends beyond biological or nutritional indicators to include psychological and social dimensions. This perspective was informed by existing literature and may have shaped interpretations. The first author maintained a reflexive journal and paid attention to potential bias during data collection and analysis. Interviews and analysis were performed by first author in collaboration with an experienced qualitative research nurse scientist; there was no previous relationships with participants.

### 2.5. Participants and Setting

Participants were recruited in person from a local community center and a church in Northeast Florida. These settings were selected because they were easily accessible and were identified as gathering places for community-dwelling older adults who are actively engaged in social activities, aligning with the study’s focus on independently living individuals. These environments offered a naturalistic context for exploring vitality as it is experienced and understood in everyday life outside of institutional settings.

Flyers were distributed in person and included a QR code linking to an eligibility survey with demographic questions and the Social Readjustment Rating Scale (SRRS) [23]. Of 12 eligible individuals, 10 community-engaged older adults completed interviews (Table 1).

A purposive sampling approach with elements of convenience and maximum variation [24] was used. Sample size was guided by principles of information power and thematic sufficiency rather than a predetermined numerical target. This study had a narrow and focused aim to understand perceptions of vitality among independently living older adults, the study applied an established theoretical framework (SDT), recruited participants with relevant experiences, and generated sufficient interview data to conduct in-depth thematic analysis.

Participants were eligible if they were 60 or older (consistent with threshold used in gerontological research) [25]; lived in their own home and managed personal care activities without assistance; and were able to speak and understand English. Participants were excluded if they scored ≤ 2 on dementia screening tool (Mini-Cog) [26] or had hearing loss or difficulty speaking. These criteria ensured participants could meaningfully reflect on fluctuations and expressions of vitality in the context of independent living.

### 2.6. Data Collection

Mini-Cog was used to detect cognitive impairments in older adults and was completed in person due to difficulty completing it online. The Mini-Cog’s specificity is 73–96%, sensitivity 73–89% [26]. SRRS evaluated participants’ stress exposure to detect any participants with extremely high levels of stress [23,27]. Participants received $25 gift card for completing the interview.

Interviews were conducted by telephone or in person using an interview guide (Table 2) and lasted 13 to 38 min. The interview guide included questions to explore definitions of vitality and factors that enhance or diminish it.

Each participant was interviewed once via telephone or in person in the comfort of their own home. The first author manually transcribed and de-identified each recording verbatim within 72 h to preserve data accuracy. Verified transcripts were then imported into NVivo version 12 Pro [28] for organization and analysis.

### 2.7. Data Analysis

This study followed Braun and Clarke’s [29] reflexive thematic analysis and results interpreted through the lens of SDT. The analysis was conducted iteratively and involved multiple phases.

The first author repeatedly listened to audio recordings and read transcripts along with field notes for immersion [29]. Coding generated 211 codes, which were organized into 27 preliminary categories and iteratively refined into seven preliminary themes. An experienced qualitative researcher independently coded the first two interviews, and coding comparisons were conducted. Discrepancies were resolved through discussion to develop a coding framework [17,30]. Through ongoing analysis and team discussion, themes were collapsed and synthesized into four final themes and one overarching theme that captured the essence of participants’ experience. SDT was not used to guide coding but was applied subsequently as an interpretive framework to contextualize and deepen understanding of the findings within existing theory (Table 3).

Theoretical integration occurred throughout the analytic process. Codes and categories were interpreted in relation to SDT constructs: expressions of independence and self-direction were mapped to autonomy, descriptions of capability and adaptation to competence, and relational and social experiences to relatedness. This deductive–inductive integration ensured that findings remained grounded in participants’ narratives while being theoretically informed.

### 2.8. Rigour

To ensure the accuracy, reliability, rigor, and overall trustworthiness of the study’s findings, multiple validation strategies were employed throughout the research process. In alignment with Lincoln and Guba’s [31] framework, trustworthiness and credibility was enhanced through the use of semi-structured interviews, prolonged engagement with the data, iterative thematic coding, cross-verification of transcripts against audio recordings, and discussion of coding decisions with an experienced qualitative researcher. Member checking was conducted with two participants who reviewed and affirmed the interpretation of findings. Transferability was supported through detailed description of the participants, setting, and study context, allowing readers to assess applicability to other populations. Dependability was strengthened through collaboration with an experienced qualitative researcher and documentation of analytic decisions throughout the study. Confirmability was supported by maintaining an audit trail and reflexive journal to document methodological and interpretive decisions, helping ensure that findings remained grounded in participants’ narratives rather than researcher assumptions. Results are reported in accordance with the Consolidated Criteria for Reporting Qualitative Research (COREQ) (Table S1) [32]. Findings are presented as themes and subcategories, supported by illustrative participant quotes to reflect the depth and context of the data.

## 3. Results

### 3.1. Qualitative Findings

**Figure 1:**
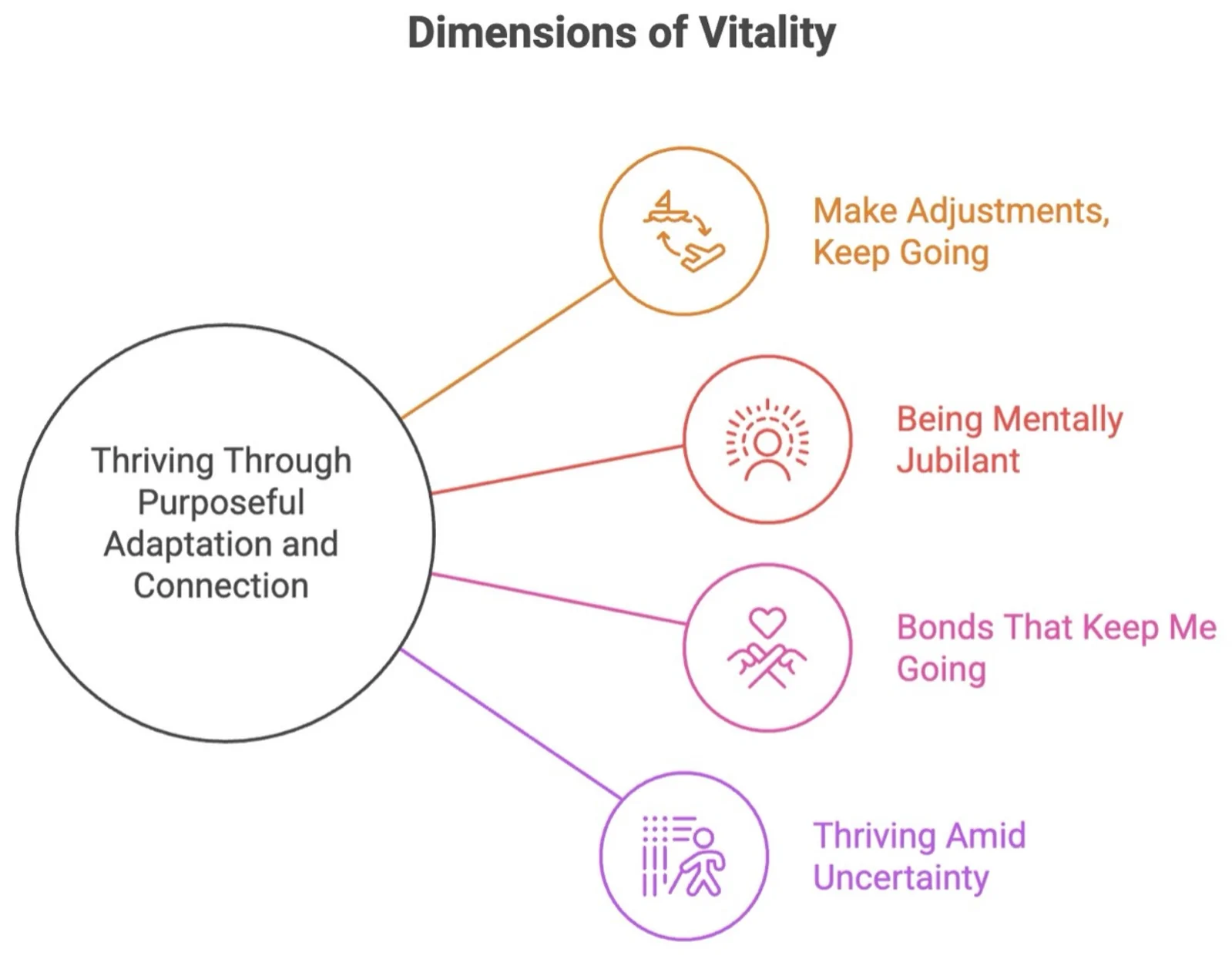
Conceptual Model: Vitality.

Participants defined vitality from their perspective, which comprised the overall theme of *Thriving through purposeful adaptation and connection in later life* (Figure 1). This theme highlighted how older adults actively navigate aging and what vitality means to them. The following four themes were identified: (1) *Make Adjustments, Keep Going*; (2) *Being Mentally Jubilant*; (3) *Bonds that Keep me Going*; (4) *Thriving Amid Uncertainty*. Together, these themes illustrated how participants in this study experience and perceive vitality that evolves as a multifaceted construct rooted in physical, emotional, cognitive, and social dimensions.

#### 3.1.1. Theme 1: Make Adjustments, Keep Going

Participants described vitality as an ongoing process of adapting to physical limitations while maintaining independence. This theme reflects two key aspects: *Acceptance and Adaptability*, where participants adjusted routines and stayed active to preserve energy, and *Nutrition and Dietary Adaptation*, which highlights how dietary choices supported vitality, even if they were not central to its definition.

**Acceptance and Adaptability:** Participants emphasized acceptance of age-related changes while continuing to engage in meaningful activities. Rather than viewing vitality as lost, they reframed it as something sustained through adaptation and persistence. They showed a determination to adapt and remain independent despite physical changes. As one participant put it, “I *might be a little slower, but I’m* [still] *going to do it*”, emphasizing the commitment to keep going and adapting as needs arise. Although participants acknowledged declines in physical ability, these changes were not viewed as barriers. Instead, they adapted to a “new normal,” recognizing limitations while continuing to remain active. One stated, “*I had more vitality than I have now, and I just think physically as you age, you lose some of that*.” Participants also acknowledged that some aspects of decline were beyond their control. As one noted, “*You can’t hang from things and do stuff like you could when you were 25*,” recognizing the inevitability of aging process and how it affects physical functions of the body.

Many participants emphasized staying physically active as a key strategy for sustaining energy and independence. They kept going to remain vital. One participant shared, “*I don’t sit around watching TV all day. I have to stay busy doing something that will give me energy*.” They often remarked that they “*feel better”* when they engaged in physical activity. Participants expressed the inevitability of aging process, but they were able to recognize their limitations and adapt accordingly.

**Nutrition and Dietary Adaptation:** Nutrition, which is the ICOPE’s proxy for vitality, was seldom mentioned by participants. However, nutrition emerged as a relevant factor of vitality when prompted by the interviewer. They knew that if they did not have a good diet that they may not be able to actively function like they wanted to. They were able to see the connection between nutrition and physical energy. One explained, “*If you don’t eat right, then your body is only going to use what you are offering it*,” meaning poor intake is associated with poor energy output. Another stated, “*Having good nutrition…sort of brings that sense of energy*.” Several participants reflected on their changing nutritional habits after noticing physical limitations. One lamented that they “*used to be able to eat anything and just go run and it was no problem*” in terms of not gaining weight. Another, recognizing the need to make adjustments due to aging, stated they changed the way they ate and how they looked at food. They consciously adapted to age-related changes. In order to maintain vitality, the participants implemented proactive nutritional strategies to accommodate aging bodies.

Overall, participants in this study described vitality as an adaptive process requiring intentional adjustments to sustain energy in the face of physical changes. Staying active was emphasized as essential to preserving energy, while nutrition emerged as a supporting factor, important but not central, to their own vitality.

#### 3.1.2. Theme 2: Being Mentally Jubilant

Another dimension of vitality involved maintaining mental engagement and a positive outlook. Participants emphasized the importance of mental energy, cognitive stimulation, and emotional awareness. Humor, optimism and gratitude were described as important ways of sustaining vitality. *Being Mentally Jubilant* reflects how participants in this study sustain mental vibrancy through everyday practices. 

**Mental Energy, Cognitive Stimulation, and Emotional Awareness:** Participants emphasized that mental energy was more important than physical energy. As one participant explained, “*I feel re-energized…mental energy is a big part of it even if physical energy is depleted*,” suggesting that staying mentally engaged can help offset physical fatigue. From participants’ perspective, vitality is not solely dependent on physical strength; rather, it includes cognitive stimulation to sustain a sense of energy.

Participants associated vitality with everyday experiences that supported curiosity and enjoyment rather than major achievements. Staying mentally active was essential: “*Keep yourself interested in things and just feel whatever you do makes you happy.*” Activities such as hobbies, laughter, and travel helped them feel vital, “*whether that’s gardening or traveling.*” Vitality was described as choosing what makes one feel mentally and emotionally alive. One participant stated they had “*a love for life and a love for experience, and a love for doing,*” indicating that involvement in meaningful activities supports a sense of being mentally energized and emotionally connected.

Whether through travel or quieter pleasures like reading, participants found cognitive engagement and emotional fulfillment in their passions. One very active participant found vitality in “*origami, playing video games, singing, dancing, and making people laugh.*” Pursuing passions with enthusiasm was often brought up in interviews, many advised, “*Find something that’s a passion for you and do it with all your heart* [and] *whatever it is that turns you on, just go for it with gusto.*” These reflections highlight how mental stimulation, curiosity, and personal expression are central to vitality. For one participant these things “*just puts something back into my soul.*”

Participants acknowledged emotional fluctuations as part of life, with some participants expressing acceptance of emotional lows. For example, one said, “*It’s okay to have feelings and to be depressed and to be down*” and another stated, “*I’m up-and-down in my emotions*” these comments reflect a realization of emotional fluctuation. These emotional shifts were not viewed as setbacks but as natural experiences to be navigated with intention. Participants’ narratives suggested that vitality could be maintained despite emotional fluctuations.

Sometimes emotional fluctuations negatively impacted participants’ vitality, particularly grief and loneliness. Emotional memories of loss significantly influenced their sense of vitality and some had to rely on medical treatment to regulate their emotions. One participant stated, “*I went on antidepressants for a year because all I did was cry*” but having close connection with friends and family helped overcome the elements of sadness and grief, “*I went out and played bridge* [with friends]” and “[having family] *that’s very important to me.*” Participants described using clinical interventions and supportive relationships to regulate these emotions and preserve mental well-being in order to sustain a mentally jubilant perspective despite experiences of loss.

**Optimism, Gratitude, and Humor:** Participants described optimism, gratitude, and humor as important ways to sustain vitality, helping them feel mentally uplifted and better able to manage their emotions. Many shared that staying positive helped them cope with health challenges, “*I have plenty of health issues that could go south, but I’m pretty optimistic about that*.” Another participant described vitality as a having a positive outlook, “*Vitality is when you look forward to each day.*” In addition, gratitude had a positive impact on vitality. Participants often expressed the importance of appreciating life and not taking it for granted. Many observed that they “*Don’t take every day for granted*” because “*life can be taken away at any moment.*” Older adults showed awareness that time is limited and it should be valued. This sense of gratitude showed that participants focus on what they still have and approach each day with appreciation, helping them remain mentally jubilant and preserve their vitality.

Humor also emerged as a valuable tool for staying mentally jubilant. “*Having a sense of humor* [and] *just laughing*” was seen as essential, offering a way to overcome any emotional challenges. Humor coupled with realistic thinking of “*keeping your expectations low, and you’re pleasantly surprised when things go well*” helped participants sustain a sense of vitality through everyday ups and downs.

Through emotional regulation, sustained mental energy, cognitive engagement, and hopeful outlook, combined with gratitude and humor, participants remained mentally jubilant even amid life’s challenges. These internal strategies were often complemented by meaningful social connections and external sources of support, which are explored in the next theme.

#### 3.1.3. Theme 3: Bonds That Keep Me Going

Vitality was deeply rooted in relationships, community involvement, spiritual connection, and the emotional support provided by the bond with both people and pets.

**Family Relationships:** Participants described vitality not merely as physical energy or health, but as a relational and socially engaged state of being. Vitality was sustained through meaningful connections and active participation in a social life, whether through family, friendships, community involvement, spiritual engagement, or companionship with pets. These forms of social engagement were not peripheral; they were central to how participants defined and experienced vitality.

Family relationships were foundational, and many participants emphasized the importance of staying connected with their immediate family. These close ties brought a lot of joy and a sense of energy as one participant noted, “*It’s just joyous when* [grandkids] *come to visit or we go there to visit*.” Whether living near family or visiting them, older adults described these bonds as essential for sustaining vitality. Several participants shared that they either moved closer to their children or they moved their older parents closer to them to help them maintain this emotional component of vitality, “*I moved my mom five minutes away from me, so I can see about her.*” This provided them reassurance and a sense of emotional security that strengthened their overall vitality.

**Companionship and Community Engagement:** Beyond family ties, friendships and informal social support also played a vital role in sustaining vitality. Participants described the reassurance of knowing help was available when needed, how fun it is to talk to friends, and having comradery. One participant believed comradery “*leads to vitality.*” Many participants felt lucky that they had friends as they felt supported and not lonely. One participant explained that it was comforting knowing “*that* [my friends] *would come to my rescue immediately, I could call upon any of them and they would do anything they could.*” These situations showed how much companionship helps to boost them mentally and to sustain vitality when interacting with others of similar age. Even casual interactions, such as shared meals or conversations, were described as energizing. Furthermore, surrounding self with positive people and staying away from negativity helped them create stronger bonds. They also feared isolation and thought it was imperative to stay connected with others as they reinforced the idea that vitality thrives in environments of positivity, support, and mutual care.

Community involvement was another powerful expression of vitality. Participants shared how community involvement helped foster vitality by creating a sense of purpose and contribution. Active engagement in volunteering at a children’s hospital, assisted living facility, organizing events, or participating in street cleanings were found emotionally and socially energizing despite physical exhaustion. One participant described how experimenting with different opportunities within their community helped them boost their sense of vitality, “*I got into community association work, street cleanings, and made friends… once I figured out how I could contribute, I felt very vital.*” These experiences among the participants illustrate how active participation in communal life and contributing to society reinforced a sense of purpose and vitality.

Participants also found companionship beyond human relationships. Daily routines with pets, such as walking the dog, offered structure and joy, further emphasizing that social engagement includes non-human relationships. Several participants shared that walking their dog led to meeting new friends, which helped them develop additional social connections and stay energized. As one participant shared, “*walking the dog…kind of been an advantage because I’ve met people in the community*.”

**Spirituality:** Spirituality and faith were deeply energizing for many participants. They described moments of profound vitality stemming from spiritual experiences and how they perceived it as empowering and life-restoring. For some, faith provided a transformative sense of renewal and strength. One participant, who was struggling with intrusive thoughts, shared, “*Jesus stepped in and cast whatever it was in me out. That was about the best energy time for me.*” Others found religion and spirituality as a reassuring and sustaining force that anchored their vitality. Morning routines of prayer and scripture readings were described as energizing and grounding. Participants emphasized an ongoing relational bond that provided emotional stability and purpose, specifying “*I lean on Jesus*” when things get hard. Grounding self spiritually linked participants to something greater than themselves or other humans, it connected them on a different emotional level perceived as deeply energizing and vital.

Participants defined vitality not just by what they could do, but by how they engaged socially, with people, communities, animals, and spiritual sources. Participants described social engagement as a defining aspect of their experience of vitality. Participants commonly linked vitality with relationships, purpose, and social connection. The importance of social connection in sustaining vitality was evident across participants’ narratives. Yet, even with strong relational ties, many acknowledged the unpredictability of life and the challenges it brings, an aspect central to the final theme.

#### 3.1.4. Theme 4: Thriving Amid Uncertainty

Participants acknowledged that aging and life transitions often brought unpredictability, yet they emphasized the importance of staying engaged and finding meaning, even when the future is unclear, as essential to maintaining vitality. Rather than being diminished by uncertainty, vitality was often expressed through persistence, openness, and a willingness to respond to change.

**Navigating Uncertainty**: Vitality, as described by participants, was not a fixed state but something sustained in the midst of life’s unknowns. Participants recognized that it may not be possible to predict or control the future, but it is possible to respond once things do occur to sustain a sense of vitality. One participant reflected on the ability to be flexible instead of resistant stating, “*You’re not going to be able to plan your future. Your future is going to happen, and you’re going to have to react to it, and that’s kind of been my life.*” One participant mentioned that balancing positive and negative experiences is important because vitality is not about eliminating uncertainty but about staying vital despite the challenges. “*Some things have been added, some things have been taken away*,” reflecting an understanding that vitality is a dynamic process involving life’s ups and downs. Participants actively recognized that vitality may fluctuate and it is part of life to navigate these changes as they come up.

Uncertainty was often accompanied by life transitions such as financial instability, housing insecurity, or unexpected caregiving responsibilities. Returning to work or experiencing loss of housing showed how life experiences beyond one’s control could introduce challenges to sustain vitality. Participants worried about having sufficient funds to last a lifetime, and some of them had to return to work. One participant reflected on how life’s instability did not diminish their sense of vitality, noting that restoring a meaningful relationship brought renewed purpose, “*I lived in my car for 2 years, and* [then] *I got back with my wife*.” Participants acknowledged that uncertainties affected their vitality but they found a way to navigate through hardship to successfully rebound.

Caregiving responsibilities also disrupted vitality when a spouse became ill and required full support, as one participant recalled, “*He was like my child, he wasn’t my husband anymore.*” However, establishing small goals helped regain a sense of vitality once they were accomplished, and the participant described how it felt great to know that progress was possible and life could still hold moments of achievement and control despite life’s uncertainties, “*It was really good, I could cry thinking about it was really good.*” Even in these difficult situations, participants expressed determination to keep moving forward, reinforcing that vitality persists when individuals remain engaged despite hardship.

**Reflection and Resilience:** Rather than isolating themselves in response to hardship, many participants demonstrated a remarkable capacity for reflection and resilience. They recognized that “*life is short*” and “*we don’t know what life has in store*,” but it was important to not despair. They chose to stay present and make the most of each day. Rather than dwelling on uncertainty, participants described focusing on maintaining relationships, engaging in meaningful activities, and appreciating small moments. One participant appreciated small certainties in life, saying “*the sun sets today and it rises tomorrow. You can count on that!*” Unpredictability was an opportunity for growth to preserve a sense of vitality. Staying connected to life, even when the future felt unclear, was seen as essential for sustaining vitality.

Participants described experiences in which uncertainty challenged vitality but also prompted reflection and resilience. Participants’ stories remind us that vitality is not just about thriving in ideal conditions, it is about staying connected to life, even when the path ahead is unclear. While uncertainty sometimes introduced anxiety or loss, it also encouraged participants to focus on what matters most. Ultimately, vitality was not defined by control or predictability, but by the ability to live meaningfully in the face of life’s unknowns.

## 4. Discussion

This study explored how independently living older adults define and experience vitality, and how life transitions enhance or diminish their sense of energy and aliveness. The four themes identified in this study, *Make Adjustments, Keep Going, Being Mentally Jubilant, Bonds that Keep me Going*, and *Thriving Amid Uncertainty*, reveal vitality as a multifaceted and dynamic experience. Participants described vitality as a dynamic interplay of physical, emotional, cognitive, and social dimensions, shaped by their ability to accept, adapt, find meaning, and stay connected.

Based on participants’ narratives, a preliminary conceptualization of vitality in this sample includes purposeful adaptation, mental engagement, meaningful connection, and resilience amid uncertainty. This definition emerged from participants’ narratives and reflected a multidimensional view of vitality grounded in older adults’ perspectives and aligns with SDT, in which vitality is supported by autonomy, competence, and relatedness. Participants demonstrated autonomy through adaptive strategies, competence through goal-setting and emotional regulation, and relatedness through meaningful social and spiritual connections.

### 4.1. Vitality as a Multifaceted Experience

Participants described vitality as feeling alive, energetic, and engaged, consistent with Ryan and Frederick’s [9] definition of subjective vitality as a conscious experience of possessing energy and aliveness, influenced by both psychological and somatic factors. Many participants emphasized that vitality was not merely physical but also mental and emotional, echoing Lavrusheva’s [33] finding that individuals may feel vital amid illness when they perceive themselves as capable and purposeful.

These findings may be interpreted through the lens of SDT, whereby participants’ descriptions of vitality were consistent with experiences of autonomy, competence, and relatedness.

### 4.2. Physical Vitality and Health Behaviors

The theme *Make Adjustments, Keep Going* highlighted how participants accepted and adapted to physical limitations while striving to maintain independence. Physical activities such as yardwork, walking, and group exercises were cited as key contributors to energy and independence, aligning with studies linking movement to enhanced vitality [20,34]. Participants acknowledged the inevitable decline in physical capacity with age and impact of illnesses, consistent with findings from de Breij et al., [35] who identified chronic illness as a predictor of diminished vitality. This reflects older adults’ competence, ability to adjust behaviors to maintain function, and autonomy, ability to remain active despite physical changes.

Within this sample, nutrition was infrequently raised spontaneously and typically emerged after interviewer prompts. This contrasts with the central role given to nutrition as a proxy within the ICOPE vitality domain [14]. Even though nutrition helps sustain physical health and mobility in older adults, it does not fully capture the broader experience of vitality [36,37].

### 4.3. Mental Vitality and Emotional Regulation

The theme *Being Mentally Jubilant* emphasized the importance of mental energy, cognitive stimulation, and emotional regulation. Participants described hobbies, travel, and creative pursuits as sources of mental energy that helped offset physical fatigue. This aligns with research and SDT showing that adaptive emotion regulation strategies protect psychological well-being [38,39] and that competence, communication, and emotional support are strongly tied to mental vitality [40,41,42,43].

Participants emphasized optimism, humor, and gratitude as tools for sustaining mental vibrancy, consistent with studies linking these traits to improved psychological health and longevity [44,45]. Cognitive stimulation through activities such as reading, puzzles, and creative pursuits has been shown to preserve cognitive function in older adults [46,47]. Grief, financial instability, and housing insecurity were described as deeply impactful. The loss of a spouse or partner was particularly emotional, with participants expressing enduring sadness and emotional depletion, consistent with literature identifying bereavement as a major threat to vitality [34,42]. Yet, even in hardship, participants demonstrated resilience and a capacity to adapt and sustain their vitality.

### 4.4. Social Connection and Relational Vitality

The theme *Bonds that Keep me Going* revealed that vitality was deeply rooted in relationships, community involvement, and spiritual connection. Participants described family ties, friendships, and even companionship with pets as essential to their sense of vitality. These findings are consistently reported in the literature, indicating that older adults perceive these connections as positively correlated with their sense of vitality [13], and that religious participation contribute positively to psychological health [43]. Participants frequently described cultivating supportive relationships and avoiding negative social influences, suggesting that relational factors were important to their experience of vitality. These findings align with research linking social support and meaningful relationships in sustaining vitality aligning closely with SDT’s relatedness construct [34,48,49].

### 4.5. Vitality in the Context of Aging and Uncertainty

The theme *Thriving Amid Uncertainty* captured how participants navigated life’s unpredictability with resilience and reflection. Aging, financial instability, caregiving responsibilities, and grief were acknowledged as challenges that could diminish vitality. However, participants demonstrated a capacity to adapt, set small goals, and find meaning even in hardship. This reflects Deci and Ryan’s [19] SDT view that autonomy and integration enhance vitality. Participants’ narratives suggest that vitality may be sustained through engagement with life despite uncertainty.

These findings align with and expand upon recent research by Jongeneelen et al., [50] who defined vitality as “having the physical, cognitive, and social capacities to do what you want to do,” (p. 8), supported by autonomy, independence, and meaning. This resonates strongly with the perspectives shared by participants in the current study, who emphasized the importance of maintaining a sense of self-control, adapting to limitations, and staying engaged with life through purposeful activity and connection. Both studies underscore that vitality is not merely a biomedical or physical construct but a subjective and functional experience rooted in personal goals, lifestyle, and social context. The emphasis on coping strategies, role models, and social networks in Jongeneelen et al.’s [50] work further supports the relational and adaptive dimensions of vitality observed in this study, reinforcing the need for holistic approaches in both research and practice.

These findings contribute to ongoing discussions of successful aging by highlighting that vitality is not solely determined by the absence of disease or physical decline. Instead, participants emphasized adaptation, meaning, and connection, suggesting that aging successfully may be more accurately defined through subjective experiences of well-being rather than objective health status alone.

## 5. Limitations

This study faced several limitations. Recruiting older adults, particularly from underrepresented groups, proved challenging despite targeted outreach efforts. Male participants were notably more hesitant to enroll than females, which may have influenced the gender distribution of the sample. While most interviews were conducted by telephone, two were held in person. During these in-person sessions, occasional disruptions from participants’ pets may have affected the flow or depth of responses. The qualitative nature of the study, along with potential social desirability bias, may have influenced participants’ responses and the interpretation of findings.

Although qualitative research does not seek statistical generalizability, the small sample limits the transferability of findings to other groups of older adults. The study’s aim and participants’ narratives provided sufficient information to explore perceptions of vitality within this sample.

The composition of the sample may have influenced the themes identified. Participants were recruited from a church and a community center, settings that attract individuals who are socially engaged and connected to community resources. Consequently, themes related to spirituality, social connection, volunteering, and community involvement may have been particularly salient within this sample. Individuals who are socially isolated, functionally limited, institutionalized, or less engaged in community life may describe vitality differently. Therefore, findings should be interpreted as reflecting the perspectives of independently living and socially active older adults rather than older adults broadly.

Despite the interviews lasting under one hour, the participants provided sufficient details to generate rich data for thematic analysis. There were no systematic differences observed between shorter and longer interviews.

Future research should further investigate vitality as a deeply personal and subjective phenomenon, recognizing that these findings represent an initial step in understanding this construct. Expanding this work to explore potential differences across sex, race and ethnicity, cultural backgrounds, and varying levels of functional dependence or living arrangements would provide a more comprehensive picture of how vitality is experienced among older adults.

## 6. Conclusions

Vitality, as described by independently living older adults in this study, is a multidimensional experience that goes beyond physical energy. It includes emotional resilience, mental engagement, and social connection. Participants described responding to aging, loss, and other life transitions through adaptation and purposeful engagement.

These findings suggest that participants experienced vitality as dynamic and closely aligned with the SDT concepts of autonomy, competence, and relatedness. To assess vitality meaningfully, clinical and research approaches must look beyond physical health or nutrition alone. Future assessment approaches may benefit from incorporating emotional well-being, personal purpose, and social engagement alongside physical indicators. As an exploratory qualitative study, these findings contribute an initial understanding of how independently living older adults describe vitality and may inform future studies involving larger and more diverse samples. Future research should further examine and refine this preliminary conceptualization of vitality and support development of more comprehensive measures.

## Figures and Tables

**Table 1:** Demographic characteristics.

Characteristic	*n* = 10
Age, mean ± SD (range)	72.3 ± 9.1 (61–83)
Sex, n (%)	
Female	7 (70)
Male	3 (30)
Race, n (%)	
Black or African American	2 (20)
White	8 (80)
SRRS score, mean ± SD (range)	88.2 ± 88.4 (0–280)
MiniCog score, mean ± SD (range)	4.1 ± 0.99 (3–5)

**Table 2:** Interview Guide.

Main Questions
Defining Vitality: 1.1. Describe to me what vitality means to you personally. 1.2. Describe a moment recently when you felt particularly alive or energized? 1.3. When you think about “feeling energetic” or “full of life,” what comes to mind? 1.4. How would you explain vitality to someone who has never heard the word before? Personal Experience of Vitality: 2.1. Tell me about a time in your life when you felt especially vital or full of life. 2.2. What was happening during that time? Or What do you think contributed to that feeling of vitality at that time? 2.3. Were there any people or places that played a role in that experience? 2.4. How did it make you feel physically, emotionally, or mentally? Quality of life? 2.5. How long did that feeling last, and what helped you sustain it? 2.6. How do you typically manage stress or emotional lows? 2.7. Are there any mindfulness or relaxation practices you’ve found helpful? Factors Influencing Vitality: 3.1. What role does physical health play in your sense of vitality? 3.2. How do you notice changes in your energy levels when your physical health changes? 3.3. Are there any health routines or practices that help you feel more vital? 3.4. What type of activities or practices do you believe make you feel alive or energetic? 3.5. How do social relationships (family and friends) or activities impact your sense of vitality? 3.6. Can you think of recent interaction that made you feel more alive? 3.7. How do you stay connected with others, and how does that affect your energy? 3.8. Have you found it easy or difficult to make new friends in retirement? 3.9. How important is social connection to your sense of vitality? 3.10. What type of social activities or events make you feel more alive? Or Are there any hobbies or interests that made you feel more alive? 3.11. If mentions outdoor, then ask—what role does nature or being outdoor play in your sense of vitality? 3.12. What lifestyle habits or choices influence your sense of energy or vitality? 3.13. Tell me about your living environment and how does it affect your sense of vitality. 3.14. What aspects of your home or neighborhood help you feel more alive? 3.15. What sort of changes to your environment you may have done to improve your vitality? 3.16. How do you balance rest and activity in your daily life? Life Transitions and Vitality: 4.1. Tell me how your sense of vitality has changed over the years. 4.2. How did retirement (or another major life change) affect your sense of vitality? 4.3. Are there any habits you’ve changed over the years to feel more energetic? 4.4. Are there any life stages where you felt a significant shift in your energy? 4.5. What role does sleep play in your daily energy? 4.6. What about nutrition? 4.7. Share with me some positive or negative events that affected your sense of vitality. Looking Ahead: 5.1. How do you think your vitality might change in the future? 5.2. What are you hopeful about when it comes to your vitality in the future? 5.3. What are some things that can be done to help you maintain or enhance your vitality as you grow older? 5.4. Are there any goals or dreams that keep you feeling energized? 5.5. What advice would you give to other adults your age about maintaining a sense of vitality? 5.6. What advice would you give to younger generations about maintaining a sense of vitality? 5.7. What would you like your future self to remember about staying vital? 5.8. What advice would you give to someone who feels like they’ve lost their sense of vitality?

**Table 3:** Example of Coding Framework and Theoretical Mapping to Self-Determination Theory.

Representative Quote	Code	Theme	SDT Construct
“I might be a little slower, but I’m [still] going to do it.”	Slower, but still going to do it	Make Adjustments, Keep Going	Competence
“I don’t sit around watching TV all day. I have to stay busy doing something that will give me energy.”	Don’t just sit/Stay active		Autonomy
“Mental energy is a big part of it even if physical energy is depleted.”	Mentally jubilant/Feel energized	Being Mentally Jubilant	Competence
“Keep yourself interested in things and just feel whatever you do makes you happy.”	Find what makes you happy		Autonomy
“It’s okay to have feelings and to be depressed and to be down.”	Okay to have feelings		Competence
“It’s just joyous when [grandkids] come to visit.”	Doing things with family	Bonds that Keep Me Going	Relatedness
“I moved my mom five minutes away from me, so I can see about her.”	Family		Relatedness
“I got into community association work, street cleanings, and made friends… once I figured out how I could contribute, I felt very vital.”	Community involvement/Volunteer		Relatedness
“I lean on Jesus.”	Higher being		Relatedness
“Walking the dog… kind of been an advantage because I’ve met people in the community.”	Pet companionship		Relatedness
“You’re not going to be able to plan your future. Your future is going to happen, and you’re going to have to react to it.”	Can’t plan your future	Thriving Amid Uncertainty	Autonomy
“Some things have been added, some things have been taken away.”	Some added, some taken away		Competence
“I might be a little slower, but I’m [still] going to do it.”	Slower, but still going to do it	Make Adjustments, Keep Going	Competence
“I don’t sit around watching TV all day. I have to stay busy doing something that will give me energy.”	Don’t just sit/Stay active		Autonomy
“Mental energy is a big part of it even if physical energy is depleted.”	Mentally jubilant/Feel energized	Being Mentally Jubilant	Competence
“Keep yourself interested in things and just feel whatever you do makes you happy.”	Find what makes you happy		Autonomy

## Data Availability

Due to the sensitive nature of confidential interview transcripts, raw data would remain confidential and would not be shared.

## Supplementary Materials

The following supporting information can be downloaded at: https://www.mdpi.com/article/10.3390/geriatrics11040093/s1, Table S1: COREQ Checklist.
